# Dynamic fracture regimes for initially prestressed elastic chains

**DOI:** 10.1098/rsta.2021.0395

**Published:** 2022-11-28

**Authors:** Michael J. Nieves, Pavlos Livasov, Gennady Mishuris

**Affiliations:** ^1^ School of Computer Science and Mathematics, Keele University, Keele ST5 5BG, UK; ^2^ Department of Mathematics, Aberystwyth University, Aberystwyth SY23 3BZ, UK

**Keywords:** discrete chain, dynamic fracture, bridge crack, open crack, clustering

## Abstract

We study the propagation of a bridge crack in an anisotropic multi-scale system involving two discrete elastic chains that are interconnected by links and possess periodically distributed inertia. The bridge crack is represented by the destruction of every other link between the two elastic chains, and this occurs with a uniform speed. This process is assumed to be sustained by energy provided to the system through its initial configuration, corresponding to the alternating application of compression and tension to neighbouring links. The solution, based on the Wiener–Hopf technique and presented in Ayzenberg-Stepanenko *et al*. (Ayzenberg-Stepanenko *et al.* 2014 *Proc. R. Soc. A* **470**, 20140121 (doi:10.1098/rspa.2014.0121)) is used to compute the profile of the medium undergoing failure. We investigate when this solution, representing the steady failure process, is physically acceptable. It is shown that the analytical solution is not always physically applicable and can be used to determine the onset of non-steady failure regimes. These arise from the presence of critical deformations in the wake of the crack front at the sites of the intact links. Additionally, we demonstrate that the structural integrity of the discrete elastic chains can significantly alter the range of speeds for which the bridge crack can propagate steadily.

This article is part of the theme issue ‘Wave generation and transmission in multi-scale complex media and structured metamaterials (part 2)’.

## 1. Introduction

The investigation of dynamic failure regimes in a structured medium—their type, average speed and how they manifest—is important in improving the resilience of the medium’s response to static loading and vibration. In turn, the associated studies have the potential to lead to, for instance, enhanced materials and civil engineering structures.

Analytical models characterizing so-called *steady* *fracture* processes in lattice materials have been the focus of a growing number of articles in the literature. The first of these models appeared in [1,2] in the study of a Mode III crack steadily propagating through an isotropic elastic lattice due to remote oscillations and constant loads. There the crack evolves as a result of the sequential disintegration of links located between two neighbouring rows of the lattice. Physically, the process may be regarded as the analogue of the domino effect, studied analytically in [3]. Mathematically, using the Fourier transform, the problem of steady lattice fracture can be reduced to a Wiener–Hopf equation [4], embedding a function which describes the dynamic behaviour of the medium when it fails. The work of [1,2] has been further developed in [5] to handle the propagation of Mode I and II cracks in discrete elastic structures.

Owing to recent trends in advanced materials, the approach has subsequently initiated a range of works investigating how different physics and mechanical elements influence steady failure regimes for the purpose of designing new elastic media. We mention the investigation of anisotropic [6,7] or dissimilar elastic properties in promoting steady failure processes [8–10], lattices with embedded high-contrast interfaces undergoing separation due to the action of highly localized waves [11] and the failure of materials with non-local elastic interactions [12].

Steady elastic lattice fracture has also been considered to be driven by shearing [13] and moving point forces [14]. Different mechanisms leading to steady failure processes, including those with time-incubation periods, have also been studied in [15].

**Figure 1.**
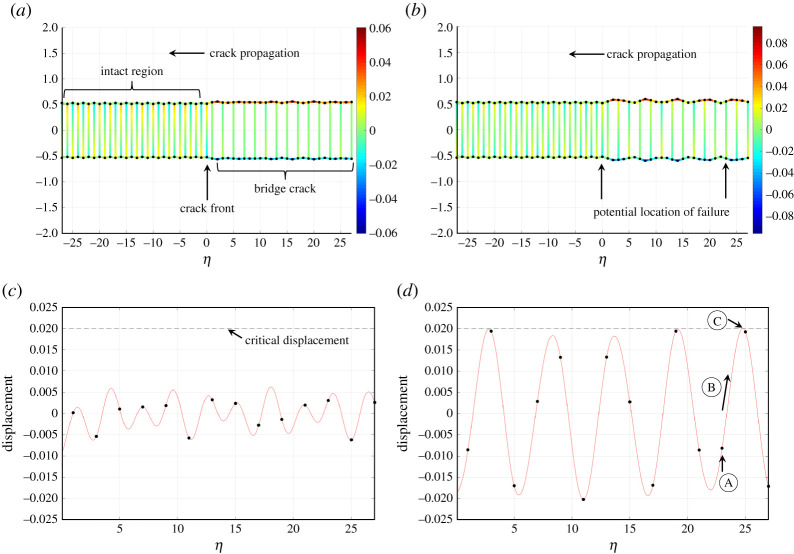
Bridge crack propagating with speed (*a*) 0.8 and (*b*) 1.395 through an isotropic chain. In this structure, the transverse links located at even and odd values of η≤0 have a length contrast of 9.16% and 11.68% in (*a*) and (*b*), respectively. The colour map represents the displacement in the structure and η is a moving coordinate used to trace the movement of the crack front. Trajectories (red curves) taken by the odd-numbered nodes (black dots) along the upper row of the medium during the failure process are shown in (*c*) and (*d*) for the speeds 0.8 and 1.395, respectively. All computations are based on the solution describing a bridge crack moving through the chain [17] that is examined here. (Online version in colour.)

There also exist other types of steadily propagating regimes, where the disintegration of lattice links can occur at a constant speed, but without the requirement that the failure happens to neighbouring lattice connections. One such process, which is the focus of the current study, is the bridge crack regime investigated in [16], where the crack propagation is due to the presence of an initial amount of internal energy stored in the system. The propagating bridge crack is represented by the sequential removal of every other lattice link between two lattice rows. Here, we study a bridge crack advancing through an elastic chain, where the internal energy is provided by a prestress. The chain is constructed from elastic links having a small contrast in length, and this contrast brings prestress to the system. An example of the bridge crack failure mode is shown in figure 1. There, the crack propagates through a chain, whose transverse connections are composed of an alternating arrangement of two types of links having slightly different lengths. The crack is formed from the removal of vertically aligned links, whose locations are associated with even integers in the medium (see horizontal axis in the figure). In figure 1*a*,*b*, this has already happened for the positive even integers located in the wake of the crack front.

The failure of beam-made systems at a uniform speed has also been treated using the Wiener–Hopf technique with a view to potential applications in designing bridges and buildings with enhanced strength. With regard to these models, we mention [18], where the failure of a bridge is sustained by gravity loading; [19,20], where the influence of thermal loads on the collapse of bridges is studied; and [21,22], where the role of vibration in promoting both steady and non-steady regimes in flexural media is analysed. We note that static cracks [23,24] and quasi-static damage propagation [25–27] in beam-made lattice systems also have great importance in improving the lattice strength in the low-frequency regime. The Wiener–Hopf problems encountered in the studies [1,2,5–16,18–22] of dynamic lattice fracture involve scalar equations whose solution is well known. However, matrix Wiener–Hopf equations, whose general solution is not known, may appear in the study of more complex fracture processes such as models for Mode III static fracture of continuous materials involving process zones [28].

The solutions to the Wiener–Hopf problems encountered in [1,2,5–16,18–22] allow one to determine the profile of the medium for any considered speed. However, as already well established in the literature, this does not necessarily mean that such a profile can physically exist [29] or is observable in simulations. In association with this, we note that numerical studies can highlight the existence of other failure regimes not covered by the above theoretical models [17]. We also refer to the vibration-induced failure of an elastic lattice, studied both analytically and numerically in [30]. There, clustering regimes for crack growth, where a crack can grow in bursts, can be observed between any pair of consecutive stable fracture regimes. Further, regimes that mimic void nucleation and coalesence ahead of a propagating crack can be found in continuous [31] and discrete beam [32] systems.

On the other hand, the analytical solution to the Wiener–Hopf equations found in lattice fracture problems can be used to ascertain when stable regimes for crack growth exist. Those solutions failing all criteria required for this steady phenomenon to be sustained indicate the possibility of non-steady failure regimes. With this in mind, the stability of Mode III crack growth in a square cell lattice was recently revisited in [33]. Such an analysis, however, cannot reveal the type of irregular crack growth achieved, and one must resort to a transient study of the crack growth to determine this.

Here, we consider an alternative steady fracture regime that occurs within an anisotropic chain. In this special fracture regime, the failure is not sustained by the presence of an external load. It is initiated by the presence of a critical level of internal energy supplied to the system by an initial self-equilibrated static stress [34]. Under specific conditions, the energy the medium possesses can initiate and sustain the motion of a bridge crack with a uniform speed. The mathematical analysis of this phenomenon presented here may help to describe the properties and forms of travelling waves propagating through so-called ‘stick bombs’ [35], which are popular fun devices formed from initially prestressed chains of woven sticks that rapidly untangle when released.

The dynamic motion of a bridge crack in an anisotropic chain was studied analytically in [36], where the solution to the problem was obtained via the Wiener–Hopf method. Our goal in the present article is to use that solution to investigate when a bridge crack propagating with a uniform speed is realizable. Further, we extend the scope of [36] by considering the structural integrity of all links composing the medium. In doing so, we are able to establish the onset of non-steady fracture regimes in addition to obtaining information on the existence of bridge crack regimes.

As an example, figure 1 shows the response of an isotropic chain undergoing failure with certain speeds. The solution from [36] allows one to reconstruct the profile at the moment of fracture. This is assumed to occur at the crack front represented by the vertical link at η=0 in figure 1. In [36], the solution is developed assuming that only those vertical links located at even values of η may fail. The features of the two profiles shown in figure 1*a*,*b* look similar, and under these assumptions both regimes are physically realizable.

However, if one accounts for the behaviour of all vertical links in the system, the conclusions change dramatically. In figure 1*c*,*d*, we display the trajectories of the nodes located at odd η behind the crack front. These nodes are connected to the tips of the intact vertical links located there. Here, as shown by figure 1*c*, the displacement of these nodes remain below the critical value corresponding to the failure of the vertical links. On the other hand, figure 1*d* shows that the node at η=23 (located at point A) behind the tip can also reach the critical displacement. As shown by point B there, this node will follow the red curve as the crack advances to the left in the medium until it reaches the critical displacement at point C. At this point, the vertical link associated with the node at η=23 fails. Thus, for the case shown in figure 1*b*, there are two sites within the structure that can fail, and this violates the conditions under which the bridge crack can propagate steadily. In fact, as we show below, if one also considers the elongation of the horizontal links in this isotropic medium, depending on their strength, one can show that none of these regimes is physically acceptable.

The structure of the article is as follows. In §2, we introduce the problem and the governing equations of a discrete elastic chain separating as a result of the propagation of a bridge crack. In §3, we use the dynamic Green’s function for the intact system to represent the solution to the main problem. This is exploited in §4 to obtain a Wiener–Hopf equation that allows one to fully solve the considered problem and determine the profile of the medium undergoing failure. In §5, we use the analytical solution to determine physically acceptable solutions connected with a steadily propagating bridge crack. Additionally, we introduce some aspects that allow us to take into account the structural integrity of the entire medium when undergoing failure. We show that this leads to a maximum speed for bridge crack regimes and sheds further light on whether these regimes are physically acceptable. Lastly, in §6, we give some discussion and future perspectives on the study presented.

## 2. Description of the problem

### (a) Description of the geometry

**Figure 2.**
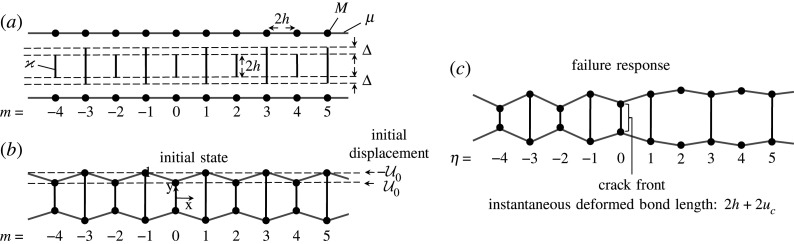
(*a*) The pre-constructed chain. (*b*) The deformed initial state. (*c*) An illustration of the bridge fracture process.

We consider two identical elastic chains, formed from links of length 2h and having stiffness μ that interconnect periodically placed point nodes with mass M at x=2hm, m∈Z (figure 2*a*). The chains are attached to each other in parallel via two types of transverse links, all having stiffness ϰ at x=2hm, m∈Z. The transverse links attaching nodes associated with an even index m have length 2h. Those attaching odd-numbered nodes are slightly longer and have length 2h+2Δ, where Δ/h≪1.

### (b) The initial configuration of the structure

When the elastic chains are connected, the contrast in the lengths of the vertical links induces statically self-equilibrated stresses in the system. The stresses are applied symmetrically with respect to y=0 (figure 2*b*) and contribute to forming the initial configuration of the medium. Correspondingly, the stresses produce the following initial displacements:

2.1 $$u_{m}(0)={\left(-1\right)}^{m}U_{0},\;m\in Z,$$

where $u_{m}(t)$, t≥0, describes the displacement of the mth node along the upper elastic chain in time and $U_{0}$ is the magnitude of the initial displacement. The latter can be determined in terms of the extension Δ as

2.2 $$U_{0}=\frac{\Delta }{2+\alpha },\;\text{where}\;\alpha =\frac{\varkappa }{\mu };$$

see [34,36].

### (c) The fracture process

We are interested in the existence of a particular steady-state failure regime caused by this initial deformation and represented by removal of sequential vertical links associated with even-numbered nodes inside the structure, forming the so-called *bridge crack*. The removal of these links occurs when a critical displacement $u_{c}$ is reached at the tip of the bridge crack located at $2hm^{*}$, where $m^{*}$ is the index associated with the bridge crack tip moving with speed v in the medium. Here, $m<m^{*}$ corresponds to the intact region, whereas $m>m^{*}$ is associated with the bridge crack. We use the moving coordinate

2.3 η=t−τm,

where τ=4h/v, to trace the position of the crack tip, corresponding to η=0. In figure 2*c*, the bridge crack is located at η>0, whereas η≤−1 corresponds to the intact part of the chain structure during this special failure regime. When the steady bridge fracture process is fully established, we also assume that all generalized coordinates describing the motion of the system are functions of η only; see figure 2.

Additionally, in [36], the theoretical model is developed under the assumption that

2.4 $$u_{2m}(t)<u_{c},\;m<m^{*},\;m\in Z,\;t\geq 0,$$

which is analogous to the Marder–Gross condition [29]. This implies that the even-numbered vertical links do not meet the failure criterion during the propagation of the bridge crack. Of course, physically, and as shown numerically in [36], such a condition cannot be guaranteed to be satisfied for every possible crack speed v. A solution to the problem we study here that satisfies (2.4) is considered to be *admissible* and realizable in, for instance, transient studies of this problem.

### (d) Energetic considerations

In studying the bridge fracture process, it is useful to compare the energy $E_{c}$ required to sustain the growth of the crack in the manner described above, i.e. the energy required to break the link at η=0, with the energy $E_{0}$ stored in this link in the initial configuration. They are given by

$$E_{c}=h\varkappa \int_{0}^{2h}{\left(W^{\prime }(\tilde{y})\right)}^{2}\;d\tilde{y}=2\varkappa u_{c}^{2}\;\mathrm{and}\;E_{0}=h\varkappa \int_{0}^{2h}{\left(W_{0}^{\prime }(\tilde{y})\right)}^{2}\;d\tilde{y}=2\varkappa U_{0}^{2},$$

where $W(\tilde{y})=u_{c}h^{-1}\tilde{y}-u_{c}$ and $W_{0}(\tilde{y})=U_{0}h^{-1}\tilde{y}-U_{0}$, $0\leq \tilde{y}\leq 2h$, are the displacements along the vertical link at the failure front during the fracture process and in the vertical links associated with even-numbered nodes in the initial state, respectively. To describe the comparison between these energies, later we use the contrast parameter

2.5 $$\gamma =\frac{E_{0}}{E_{c}}=\frac{U_{0}^{2}}{u_{c}^{2}}$$

to understand how the initial state of the system promotes this specific failure process.

Before moving on, we note that the considered failure regime and associated analysis do not completely describe how the medium would fail. Indeed, during the bridge crack advancement, only the integrity of the vertical links in the medium is considered at prescribed points, and these links fail within fixed time periods. In general, several non-steady or non-regular failure processes can occur, where the critical displacement may be achieved at any time and location corresponding to the position of intact vertical links. Additionally, the strength of the horizontal links will play a role in the material’s failure, providing new potential regimes of possible fracture. Some of these regimes are discussed below in connection with the computations presented here that are based on the analytical procedure of [36].

#### (i) Governing equations of the medium

During the bridge fracture process, the junctions along the upper row of the system are assumed to undergo motion according to the following equation from [36]:

2.6 $$M{\ddot{u}}_{m}(t)-Q_{m}(t)=\mu (u_{m+1}(t)+u_{m-1}(t))-2(\mu +\varkappa )u_{m}(t),$$

where $Q_{m}(t)$, m∈Z, is an internal forcing term taking into account the fact that behind the crack tip only the vertical links associated with odd-numbered nodes supply longitudinal forces to the masses along the upper row. This term has the form

2.7 $$Q_{m}(t)=\left\{ \begin{array}{ll} 2\varkappa u_{m}(t), & m>m^{*},\;m\;\text{even},\\ 0, & \text{otherwise.} \end{array}\right.$$

Here, $m^{*}$ denotes the position of the failure front. Additionally, the form of $Q_{m}(t)$ follows from the assumption that the system’s displacements are symmetric relative to y=0. The displacements $u_{m}(t)$ are assigned the following representation, which depends on the node location in the medium:

2.8 $$u_{m}(t)=U_{m}(t)+{\left(-1\right)}^{m}U_{0},\;m\in Z.$$

The function $U_{m}(t)$ describes the perturbation of the displacements of even- and odd-numbered nodes from their initial state in (2.1). Recall that the time-independent terms in the above satisfy the static equations of equilibrium (compare with (2.6) with a zero left-hand side). Going forward, we assume that $U_{m}(t)$ takes the form

$$U_{m}(t)=\left\{ \begin{array}{ll} U(\eta ) & \text{for}\;m=0,\pm 2,\pm 4,\pm 6,\ldots ,\\ V(\eta ) & \text{for}\;m=\pm 1,\pm 3,\pm 5,\ldots , \end{array}\right.$$

where U and V are two separate functions depending on the moving coordinate η. In addition, owing to this change of variable, we have

2.9 $$Q_{m}(t)=Q(\eta )=\left\{ \begin{array}{ll} 2\varkappa (U(\eta )+U_{0}), & \;\eta >0,\;\eta \text{ even},\\ 0, & \;\text{otherwise.} \end{array}\right.$$

## 3. Intermediate solution in terms of dynamic Green’s functions

The displacements U(η) and V(η) are determined via a double convolution with respect to m and t, which uses the transient Green’s function for the dynamic problem of the intact chain. Following [36], we have

3.1 $$U(\eta )=\sum_{\tilde{m}=0,\pm 2,\ldots }G(m-\tilde{m},t)*Q(t-\tau \tilde{m})\;\text{for }m=0,\pm 2,\pm 4,\ldots$$

and

3.2 $$V(\eta )=\sum_{\tilde{m}=0,\pm 2,\ldots }G(m-\tilde{m},t)*Q(t-\tau \tilde{m})\;\text{for }m=\pm 1,\pm 3,\ldots ,$$

with the ∗ denoting convolution in time. Here, we recall that τ represents the time period between the removal of even-numbered vertical links in the medium (see (2.3)). Additionally, G solves (2.6) with $Q_{m}(t)$ replaced by $\delta (t)\delta_{m,0}$, where δ(t) is the delta function in time and $\delta_{m,0}$ is the Kronecker delta

3.3 $$\delta_{m,n}=\left\{ \begin{array}{ll} 1 & \text{if}\;m=n,\\ 0 & \text{otherwise}. \end{array}\right.$$

One can then apply the Fourier transform with respect to η, denoted by a superscript F, to representations (3.1) and (3.2). Using the fact they involve convolution-type integrals and summation operations, they can decomposed into a product of transforms with respect to t and m. As such, via appropriate changes of variable one obtains that the transformed functions have the forms

3.4 $$U^{F}(k)=Q^{F}(k)G_{\text{even}}(k)\;\text{and}\;V^{F}(k)=Q^{F}(k)G_{\text{odd}}(k).$$

Here, the transformed functions are defined as

$$\{U^{F}(k),V^{F}(k),Q^{F}(k)\}=\int_{-\infty }^{\infty }\{U(\eta ),V(\eta ),Q(\eta )\}\;e^{i\eta k}\;d\eta ,$$

and we have

$$G_{\text{even}}(k)=\frac{c^{2}}{\mu }\frac{{\left(0-ik\right)}^{2}+2c^{2}(\alpha +1)}{{\left[{\left(0-ik\right)}^{2}+2c^{2}(\alpha +1)\right]}^{2}-4c^{4}\cos^{2}(\tau k)}$$

and

$$G_{\text{odd}}(k)=\frac{c^{4}}{\mu }\frac{2\cos(\tau k)}{{\left[{\left(0-ik\right)}^{2}+2c^{2}(\alpha +1)\right]}^{2}-4c^{4}\cos^{2}(\tau k)},$$

with $c=\sqrt{\mu /M}$. Note that $G^{F_{m}F_{t}}(-\tau k,k)=G_{\text{even}}(k)+G_{\text{odd}}(k)$, where $F_{m}$ and $F_{t}$ denote the discrete and continuous Fourier transforms with m and t, respectively.

## 4. The Wiener–Hopf equation and its solution

The relation (3.4)_1_ is independent of (3.4)_2_. Consequently, this can used to identify $U^{F}(k)$ and then $V^{F}(k)$. By using (2.9), we have

4.1 $$Q^{F}(k)=2\varkappa \left(U^{F}(k)+\frac{U_{0}}{0-ik}\right),$$

and upon insertion of this into (3.4)${\;}_{1}$ one arrives at a scalar Wiener–Hopf equation for $U^{F},$

4.2 $$U_{-}(k)+L(k)U_{+}(k)=[1-L(k)]\frac{U_{0}}{0-ik},$$

where

$$L(k)=1-2\varkappa G_{\mathrm{even}}(k)$$

and the standard representation for $U^{F},$

4.3 $$U^{F}(k)=U_{+}(k)+U_{-}(k),$$

has been used, with the subscript + (−) indicating a function that is analytic in the upper (lower) half of the complex plane for k∈C. The kernel function L(k) corresponding to this equation possesses the following split:

L(k)=limIm k→0L+(k)L−(k),with L±(k)=exp⁡(±12π∫−∞∞ln⁡L(ξ)ξ−k dξ), ±Im k>0.

Here, the functions $L_{\pm }(k)$ satisfy the conditions

4.4 $$\lim_{k\to \pm i\infty }L_{\pm }(k)=1\;\text{and}\;L_{\pm }(0)=\frac{R^{\pm 1}}{\sqrt{\alpha +2}},\;\text{where}\;R=\exp\left(\frac{1}{\pi }\int_{0}^{\infty }\frac{\text{Arg}\;L(\xi )}{\xi }\;d\xi \right).$$

Following [36], (4.2) is then readily solvable and one has

4.5 $$U_{+}(k)=\frac{U_{0}}{0-ik}\left[\frac{1}{L_{-}(0)L_{+}(k)}-1\right]\;\text{and}\;U_{-}(k)=\frac{U_{0}}{0-ik}\left[1-\frac{L_{-}(k)}{L_{-}(0)}\right].$$

Note that as in [36], one can compute particular limits of the preceding functions that provide information about the physical response of the medium at selected points. In particular,

$$U(0)=\lim_{k\to -i\infty }ikU_{-}(k)=\lim_{k\to i\infty }(-ik)U_{+}(k)=U_{0}\left(\frac{1}{L_{-}(0)}-1\right),$$

and recalling (2.8), we deduce that the critical displacement achieved at η=0 during the bridge crack advancement is $u_{c}=u(0)=U_{0}/L_{-}(0)$. Thus, using (2.2) and (2.5) provides a relationship linking the crack speed v with the energy contrast ratio γ:

4.6 $$\gamma ={\left(L_{-}(0)\right)}^{2}=\frac{1}{(2+\alpha )R^{2}},$$

where (4.4) was employed in deriving the last equality. As shown in [36] and discussed below, for a given γ, the preceding relation can be satisfied for several values of the speed v, and only some of these speeds correspond to a bridge fracture process that is admissible. The combination of (4.5) through (4.3) and the use of the result in (3.4) together with (4.1) leads to explicit expressions for the functions $U^{F}$ and $V^{F}$. The actual displacements are then computed using the inverse transform on k as

4.7 $$U(\eta )=\frac{1}{2\pi }\int_{-\infty }^{\infty }U^{F}(k)\;e^{-ik\eta }\;dk\;\text{and}\;V(\eta )=\frac{1}{2\pi }\int_{-\infty }^{\infty }V^{F}(k)\;e^{-ik\eta }\;dk.$$

## 5. Numerical computations: admissibility of identified regimes

Here, we investigate the behaviour of the analytical solution (see (2.8), (4.7), (3.4), (4.3) and (4.5)) and use this to ascertain information concerning the admissible bridge crack regimes.

### (a) Numerical settings and admissibility criteria

#### (i) Computational configuration

We consider three different structures formed from nodes of mass M=1 kg and links of length 1 m (h=0.5 m), with horizontal links having stiffness μ=1 Pa. The vertical members have different stiffnesses characterized by α=0.2,1 and 5.5. The propagation of the bridge crack is assumed to occur when the vertical link at the front of this process reaches a critical elongation defined through $u_{c}=0.02$.

**Figure 3.**
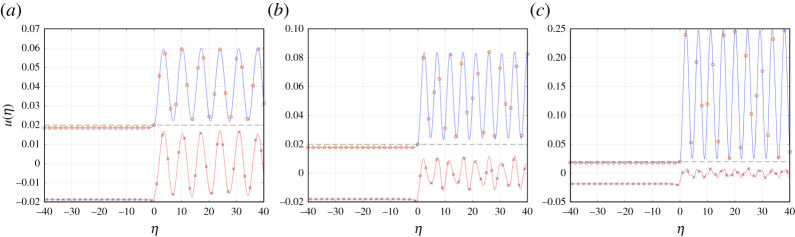
Node trajectories for v=1.1 and (*a*) α=0.2 (Δ=0.0409), (*b*) α=1 (Δ=0.0539) and (*c*) α=5.5 (Δ=0.1368). The trajectories of the even-numbered nodes (shown as red circles) are depicted by blue curves, whereas the odd-numbered nodes (blue crosses) will follow the trajectories shown as red curves. (Online version in colour.)

For a given structure, we perform a parametric study of the influence of v on the behaviour of the structure’s profile, defined via (2.8) and (4.7). The speed v is used to calculate the values of γ and Δ, through (2.2) and (2.5), needed to sustain the propagation of the bridge crack. We recall that Δ defines the initial configuration for the system through the displacement $U_{0}$ (see (2.1) and (2.2)) that leads to a bridge crack propagating with speed v in the steady-state regime. Additionally, the quantity $U_{0}$ is needed for the computation of the structure’s profile via (2.8), which is based on (4.7) together with (3.4), (4.3) and (4.5). We note that the numerical inversion of the Fourier transform is performed in (4.7). Figures 3 and 5–7 below present an analysis of the medium’s profile during the fracture process, and this has been constructed for the nodes within the interval |η|≤40.

Note that the procedure outlined above has been used to compute the profiles in figure 1, which are admissible under the framework described in [36], but based on the analysis presented below these regimes may not be physically realizable.

#### (ii) Admissibility conditions: failure of even-numbered vertical links

We recall that for a given speed v, as in [36], an admissible failure regime is defined in accordance with condition (2.4), which takes into account the elongation of even-numbered vertical links ahead of the failure front only. Following the computation of the profile, we will apply this admissibility condition to deduce ranges of the speed v for which the bridge crack can propagate steadily.

#### (iii) Failure of horizontal links and odd-numbered vertical links

Further, as mentioned earlier, the structure can fail in several other ways. In addition to the possible failure mechanism incorporated into the admissibility condition (2.4), potential sites of failure could occur with the horizontal links and vertical links associated with odd indices. The analytical solution (see (2.8) and (4.7)) is not applicable to describing the resulting dynamic failure regimes, but it can be used to predict the instances where these regimes initiate. Consequently, we introduce some quantities to study these alternative failure mechanisms. These quantities include

5.1 $$\beta =\max_{\eta \in R}\frac{-U_{0}+V(\eta )}{u_{c}}\;\text{and}\;\varpi =\frac{1}{2u_{c}}\max_{\eta \in R}\left(\sqrt{{\left(\alpha U_{0}+V(\eta )-U(\eta )\right)}^{2}+4h^{2}}-2h\right),$$

which enable us to check the structural integrity of the chain at odd-numbered vertical links (see (2.8)) and of the horizontal links, respectively. Here, the expression to be maximized in ϖ is the expression for the extension of the horizontal links that connect the tips of links having two lengths 2h and 2h+2Δ. Additionally, note that β and ϖ are independent of the strengths of the odd-numbered vertical links and the horizontal links. The values of the parameters β and ϖ should be compared with the ratios

5.2 $$\beta_{0}=\frac{u_{c}^{\text{o}}}{u_{c}}\;\mathrm{and}\;\varpi_{0}=\frac{u_{c}^{\text{h}}}{u_{c}}$$

involving the strengths of odd-numbered vertical links $u_{c}^{\text{o}}$ and horizontal links $u_{c}^{\text{h}}$ to ascertain whether a steady bridge fracture regime is physically feasible or not. In a majority of the computations below, we will assume $\beta_{0}=1$ and $\varpi_{0}=1$, implying that all links have the same strength. However, it is realistic to consider links with contrasting strengths, and below we also show that this can influence the admissibility of steady failure regimes.

To compute β and ϖ, we combine the above definitions with the displacements of individual nodes found with (2.8) and (4.7) during the steady-state failure regime. In connection with (5.1)${\;}_{1}$ we also introduce the index $m_{\beta }$, which represents the index of the node where β occurs.

### (b) Trajectories of the nodes based on the analytical solution

Here, we give some examples of the trajectories taken by the nodes of the medium during the bridge failure process. The trajectories are based on the solution (2.8) that uses (4.7), (3.4), (4.3) and (4.5). We determine the trajectories as functions of the continuous variable η. The profiles obtained allow us to accurately capture when and where the structure can fail as time elapses in the steady-state failure process for a given bridge crack speed.

First, we discuss the features of a typical admissible failure mode according to (2.4). The profiles of these admissible regimes for α=0.2,1 and 5.5 are shown in figure 3. There, the trajectories of the even- and odd-numbered nodes are shown as continuous curves. During a single time period τ of the bridge crack propagation, all nodes will move along these trajectories two lattice units to the right. As a result, we note that the maximal displacement of each node may be achieved at some time during the movement of the node along these curves. An example includes the node indexed as η=3 along the red curve in figure 3*a,* whose maximum displacement occurs for a non-integer value of η.

As expected, figure 3*a*–*c* shows that all displacements for η<0 do not exceed $u_{c}$, represented by the horizontal dashed lines in these plots. In fact, the displacements of even nodes are approximately uniform along the upper horizontal row of the chain when η<0. The same is true for the odd-numbered nodes, which have displacements of approximately the same magnitude but with opposite sign. The result implies that the structure possesses an alternating strain deformation ahead of the bridge crack front [36]. In all admissible regimes computed below, this behaviour located at η<0 is typical during the bridge fracture process. In fact, in the next sections, we demonstrate that it is the behaviour of the structure within the bridge crack zone at η>0 that provides an upper speed limit for this failure regime.

Additionally, the analytical model developed above assumes $u(0)=u_{c}$, which is the case for all trajectories shown in figure 3. For η≥1, the trajectories undergo oscillations. This results from the release of waves during the bridge fracture process that travel outward to the right of the front. In relation to this, as demonstrated in figure 3, for η>0 the displacement amplitude of even-numbered nodes can increase with increasing α, whereas the displacement amplitude of the odd-numbered nodes will decrease. Physically, this observation corresponds to the fact that stiffer vertical links will prohibit the odd-numbered nodes for η>0 from undergoing large displacements, whereas the less constrained even-numbered nodes in this region will be more prone to excitation.

### (c) Energy ratio dependency on the bridge crack speed

**Figure 4.**
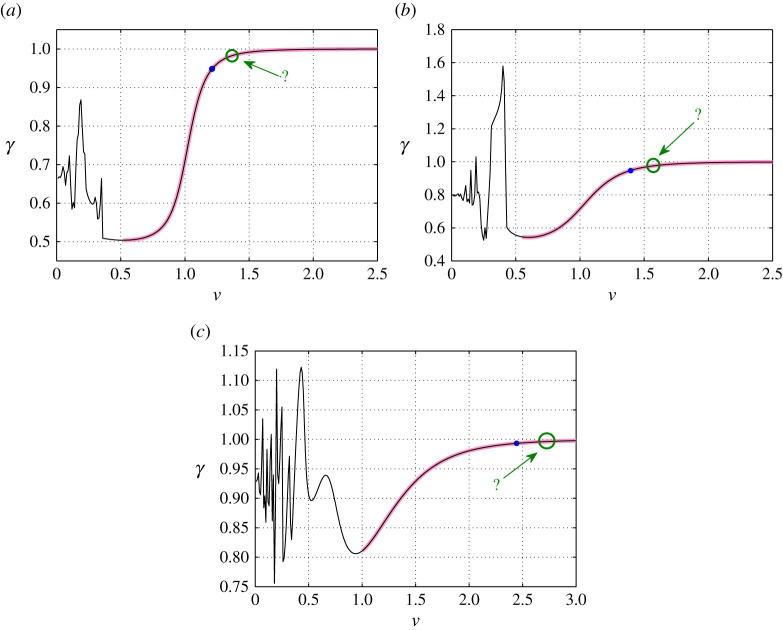
The energy ratio γ as a function of the bridge crack speed for a structure with α=0.2,1 and 5.5. Computations are based on (4.6). Red segments of each curve indicate intervals of the speed v for which the analytical solution (see (2.8) and §4) is admissible as defined in accordance with (2.4). Blue dots represent the upper bounds for the speed interval corresponding to the bridge crack regime. Green circles indicate the possible minimum speeds for the open crack regime (see discussion in §5d and the numerical results of [36] that describe the transition from the steady bridge crack regime to the open crack regime). (Online version in colour.)

In figure 4, we show the dependency of the energy ratio γ on the bridge crack speed that has been computed using (4.6) for the three structures considered.

The behaviour of γ can be described with two distinct regimes: (i) a regime where γ grows monotonically with increasing v and approaches unity when v→∞; and (ii) a regime occurring at low speeds, where the value of γ can oscillate rapidly with increasing v. For regime (i), indicated in figure 4 by red segments of the curves, the analytical solution generates an admissible profile for the bridge crack failure regime as described by (2.4). The low-speed regime (ii) widens with increasing α, and we note that the admissibility of failure modes in this regime is seldom or not possible. These conclusions are also supported by the numerical simulations presented in [36].

The blue dots shown in figure 4 represent the upper bound for the bridge crack regime within the admissible speed interval. The speeds associated with the upper limits are in good agreement with those obtained in [36] via a numerical transient analysis of the system and its failure. These maximal speeds have been determined by computing the profiles for the chain structure with the analytical solution and observing the behaviour of those vertical links not taken into account by (2.4). The role of these links in the bridge fracture process is discussed in next section.

### (d) Maximum speed for the bridge fracture regime

The odd-numbered vertical links, having the same material properties as the other vertical links in the chain, are important in determining the maximum possible speed for a bridge crack propagating in the chain. Thus we turn to analysing β for this purpose, assuming that odd-numbered links and even-numbered links have the same strength. In this case, as discussed below, the value $\beta =\beta_{0}=1$ reflects a crucial scenario in which an odd-numbered link may also fail within the system.

**Figure 5.**
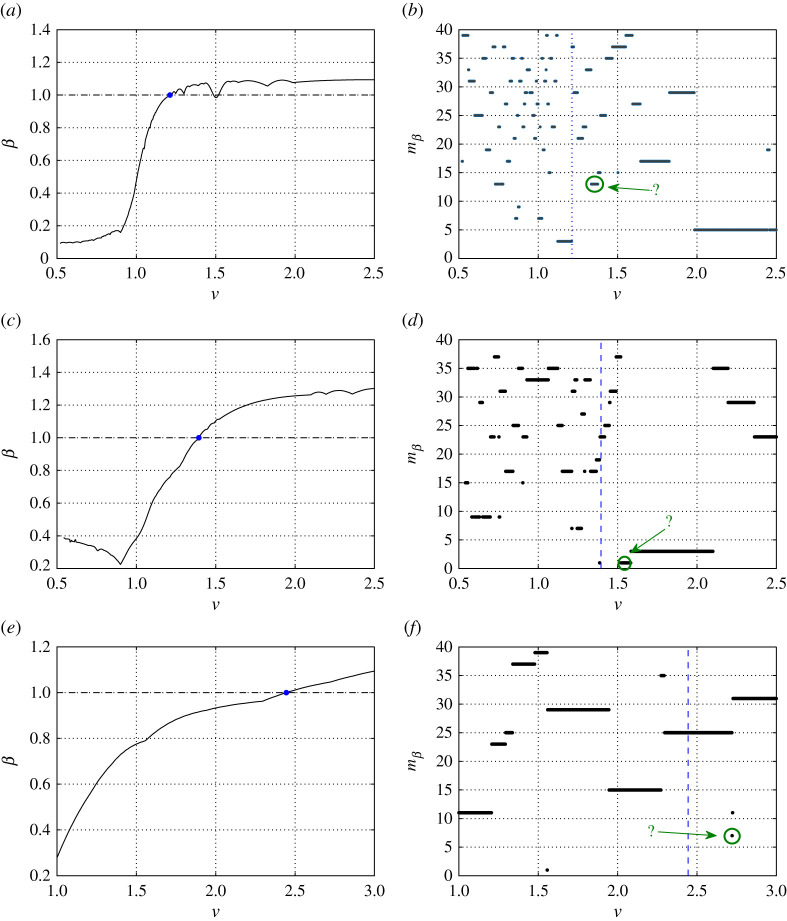
The dependency of the parameter β in (5.1) and $m_{\beta }$ on the bridge crack speed v for (*a*,*b*) α=0.2, (*c*,*d*) α=1 and (*e*,*f*) α=5.5. The critical value $\beta =\beta_{0}=1$, corresponding to the failure of an odd-numbered vertical link in the chain structure, is shown as a horizontal and vertical dashed line in (*a*,*c*,*e*) and (*b*,*d*,*f*), respectively. The blue dots in (*a*,*c*,*e*), where β=1 is reached for the first time as v is increased, correspond to those shown in figure 4. (Online version in colour.)

Figure 5*a*,*c*,*d* shows the parameter β of (5.1) as a function of the failure speed v. The results are presented for speeds v associated with an admissible profile of the medium in accordance with (2.4). Hence the horizontal axes in these figures have also been selected based on this (see also figure 4). The computations in figure 5*a*,*c*,*e* show that β is a continuous function of v and that for large α, i.e. when the vertical links are much stiffer than the horizontal ones, β behaves monotonically with respect to v. In the cases of α=0.2 and 5.5, the minimum of β occurs at the lowest admissible bridge crack speed. On the other hand, β possesses a global minimum for α=1 within the range of admissible speeds (figure 5*c*).

If we assume $\beta_{0}=1$ (see (5.2)), figure 5*a*,*c*,*e* shows that increasing the crack speed causes β to increase up to and beyond $\beta_{0}$ at a specific v. For a given speed, when $\beta_{0}$ is reached or when β>1, this indicates that there exists at least one odd-numbered vertical link in the medium that achieves an elongation equal to or greater than the critical elongation. Thus, this link will fail during the propagation of the bridge crack. This implies that the steadily propagating bridge crack is no longer feasible. The points where β reaches unity in figure 5*a*,*c*,*e* are used to define the points of the maximum speeds $v_{c}$ for the bridge crack regime, indicated by blue dots in figure 4.

Note that it follows that if the strength of the odd vertical links differs from the strength of the even vertical links, i.e. $\beta_{0}\neq 1$, the β=1 case no longer indicates the moment when an odd-numbered link can fail inside the medium. Consequently, the horizontal dashed lines in figure 5*a*,*c*,*e* would also have different locations corresponding to the value of $\beta_{0}$. The shift in the value of $\beta_{0}$ influences the interval size of the admissible speeds for steady bridge crack failure. In the cases where α=0.2 and 5.5, the global minimum of β occurs at the smallest admissible failure speed, and subsequently decreasing $\beta_{0}$ only adjusts the upper bound for the bridge crack regime. In the case of α=1, figure 5*c* shows that one can decrease $\beta_{0}$ until the lower bound for the admissible speed regime can be increased. This result is based on the presence of the global minimum of β, achieved around v=0.9 in figure 5*c*. Interestingly, if $\beta_{0}$ is below the global minima shown in figure 5*a*,*c*,*e*, then we can conclude that the steady bridge crack is never realizable for those structures considered.

In all cases, the parameter β is monotonic as a function of v in the vicinity of β=1. When α=0.2 and 1, β possesses several minima. Interestingly, in the case of α=0.2 (figure 5*a*), beyond the critical speed located at approximately v=1.22 where $\beta =\beta_{0}=1$, there is a local minimum near v=1.5, and in the vicinity of this point we have β<1. The appearance of this local minimum seemingly indicates that beyond the maximum speed for the bridge crack, there may exist some narrow speed range where this failure phenomenon will propagate steadily. We note that for α=0.2, after reaching β=1 at around v=1.22 it is clear that the steady bridge failure regime ceases to exist. After this point, the analytical model does not indicate what non-steady failure mechanisms develop from the initial strained state and their behaviour with increasing Δ. As a result, it is not evident from our analysis if one can realize those possible stable states observed in figure 5*a* in the vicinity of v=1.5. However, such states may be possible if the associated profile deformations are used to provide the initial strains to the system.

We also report the behaviour of the index $m_{\beta }$, where the maximum of β occurs, in figure 5*b*,*d*,*f*. There, the upper bound for the bridge crack regime is shown as a vertical blue dashed line. The plots demonstrate that the index $m_{\beta }$, where the maximum elongation for an odd-numbered vertical link occurs, is always behind the bridge crack tip. This is expected on physical grounds, as in this region the even-numbered vertices are less constrained owing to the formation of the bridge crack, and this allows the bridge crack zone to achieve larger displacements. Additionally, below the maximum bridge crack speed (indicated by a blue vertical dashed line), the position where β occurs can rapidly fluctuate behind the crack front if α is sufficiently low, e.g. see figure 5*b*,*d*. There, we observe that there exist narrow speed intervals where $m_{\beta }$ is fixed. When the solution is no longer physically acceptable, the index $m_{\beta }$ does not undergo rapid changes with increasing v and can remain constant for large intervals of the crack speed.

**Figure 6.**
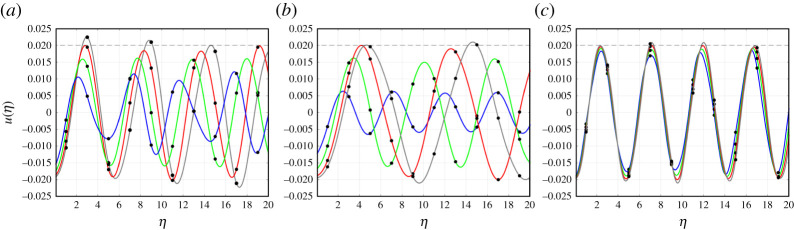
Trajectories of the nodes in the bridge crack zone (η>0). The trajectories have been computed for $v=0.8v_{c}$ (blue curve), $0.9v_{c}$ (green curve), $v_{c}$ (red curve) and $1.1v_{c}$ (grey curve), where $v_{c}$ is the maximum bridge crack speed, and for (*a*) α=0.2, (*b*) α=1 and (*c*) α=5.5. The critical displacement $u_{c}=0.02$ is represented by the horizontal dashed line. (Online version in colour.)

To further corroborate the conclusion that when $\beta =\beta_{0}=1$ for a given critical failure speed $v_{c}$ the steady bridge fracture process is not possible, in figure 6 we have computed the trajectories of the odd-numbered nodes in the medium as a function of η. There, for α=0.2,1 and 5.5, it is clear that when $v<v_{c}$ these nodes never reach the critical displacement. However, when $v=v_{c}$, the trajectory contains points where this can happen, e.g. see the red curve of figure 6*a* for α=0.2 at approximately η=2.7. One can also find similar points in figure 6*b*,*c* for α=1 and 5.5, respectively, along the red curves where $v=v_{c}$. Subsequently, as the bridge crack advances a single unit (or two lattice spacings), the node located at η=1 in figure 6*a* moves along the corresponding trajectory to the right. In following this trajectory the node will reach the critical displacement. It is important to note that this situation, in general, can occur at a completely different time from the failure event expected to occur at the crack front. The approach developed in [5,36] allows one to accurately capture the time shift between these events. At the point where the critical displacement is reached at additional sites other than the crack front, the solution describes the emergence of a non-steadily propagating crack. The beginning of this new regime signifies the end of the bridge fracture regime at $v=v_{c}$.

Additionally, beyond this critical speed the analytical solution suggests that the structure of the bridge crack may be very different. Indeed, with the presence of extra sites of fracture, a bridge crack may propagate with a structure different from that observed in figure 1 and possibly formed from a larger macro-cell of periodicity. The bridge crack is formed from a collection of periodically placed gaps behind the crack front, but beyond the maximum speed for these regimes the size and periodicity of these openings could be more complex, leading to what we term the macro-cell bridge crack. We note that the apparent structure of the openings could possibly be obtained from figure 5, which shows how the location of the maximum displacement at odd-numbered links migrates through the medium with increase of the failure speed. There, for β≥1, whenever this position changes, we obtain information about the new regions behind the crack that can reach the critical displacement. This could be connected with new openings within the medium and different bridge crack regimes with larger macro-cells. Note that within this regime, what we refer to as a fracture macro-cell is formed. By this we mean a sequence of fracture events involving a collection of vertical links that repeats in time. The structure of the finite bridge crack can then vary with increasing *γ*. Further, the sequence of fracture events can be non-monotonic with respect to the index *m*. The average speed of such a failure mode is directly related to the length of the fracture macro-cell and the times associated with the fracture of each link. In other words, the average macroscopic fracture speed is determined by the movement of the fracture macro-cell. Moreover, the sites of failure may accumulate within the medium to the point where a fully *open crack* regime can occur.

### (e) Failure within the horizontal links

In considering realizable fracture regimes, one must also take into account the deformations that the horizontal links within the medium can undergo. Using ϖ in (5.1), we can get a better understanding of their tensile motion during the propagation of the bridge crack. Once more, we focus on the case where the links in the structure have the same strength and indicate possible outcomes associated with when this is no longer assumed to be the case. Consequently, here ϖ being equal to unity will indicate a regime where horizontal links in the medium can fail in addition to even-numbered vertical links in the medium.

**Figure 7.**
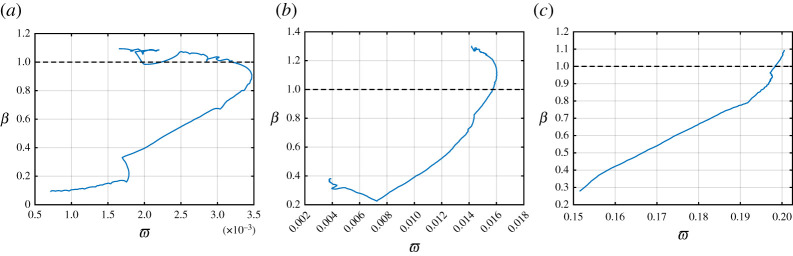
The parameter β as a function of ϖ for (*a*) α=0.2, (*b*) α=1 and (*c*) α=5.5. The computations are based on (5.1). In each diagram, the horizontal dashed line β=1 corresponds to the failure of odd-numbered vertical links and the upper limit of the bridge crack regime.

In figure 7, we plot β as a function of the parameter ϖ for α=0.2,1 and 5.5 and for only those speeds v where the bridge crack propagation is admissible according to (2.4). We note that β is a continuous function of ϖ but not injective in some regions of the curves shown.

As β approaches unity in figure 7, we see that ϖ also increases. Consequently, we can conclude that increasing the speed of the bridge crack brings increasing stresses to the entire region located behind the crack front.

In addition, the magnitude of ϖ is much smaller than that of β. This is expected on physical grounds, as the horizontal links respond to the linear vertical deformation of the transverse links, which have a small contrast in length dictated by Δ. A small vertical deformation therefore does not produce a large extension in the horizontal links. Also, as the anisotropy parameter α increases, figure 7 shows that the magnitude of ϖ increases. This result is due to the fact that increasing α corresponds to a softening of the lateral links in the medium, enabling the horizontal links to undergo greater axial deformations.

It can also be clearly seen from figure 7 that for all admissible speeds of bridge crack failure the parameter ϖ is less than 1. As a consequence, if the horizontal links inside the chain are assumed to have the same strength as the vertical links, all admissible bridge crack regimes are realizable in the structures considered.

Here, our conclusions are based on the fact that all links in the structure possess the same strength. The results leading to these evaluations use the solution from [36], which gives the profile of the chain during steady bridge failure regimes. These profiles are obtained independently of any information on the behaviour of vertical odd-numbered links and horizontal links through, for instance, β and ϖ, respectively. Of course, it is immediately clear that in all cases considered, assuming that the horizontal links have a much lower strength than the even-numbered vertical links would reduce the possibilities for potential steady bridge crack regimes to exist. Figures 5–7 then become essential in identifying the existence of the emerging failure regimes when stable bridge crack regimes are no longer supported by the system.

**Figure 8.**
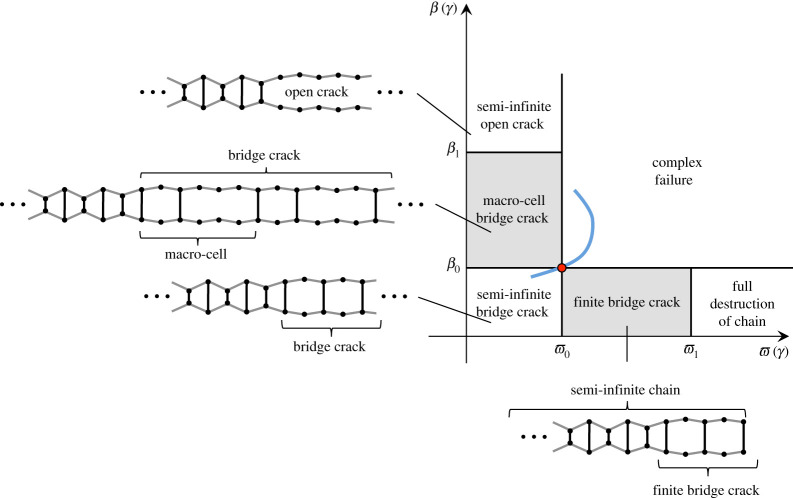
The β–ϖ plot illustrating possible subdomains of different failure regimes of the chain. The blue curve represents possible data obtained from the solution in [36] in a similar way to the computations produced in figure 7. The red dot along this curve represents the data for a given energy ratio γ. This point may reside in one of the regions defined by the values $\varpi_{j}$ and $\beta_{j}$, j=0,1, indicating when some steady failure regime or possible non-steady regime can propagate in the chain. Note that within the intermediate regions $\beta_{0}<\beta <\beta_{1}$ and $\varpi_{0}<\varpi <\varpi_{1}$, the structure of the bridge crack and its associated failure mechanism are unknown, and this requires an analysis based on features of the analytical solution described here combined with a transient study of the system. (Online version in colour.)

We discuss these possible regimes with the help of the illustration in figure 8. There, β and ϖ being equal to unity represents the failure of the medium at vertical odd-numbered links and horizontal links, respectively, in the case where the strength in the structure is uniform. However, when the strength of these links differs from the strength of the even links, we obtain new values $\beta_{0}$ and $\varpi_{0}$ where failure can occur (see also (5.2)). It is clear then that if $\beta_{0}>1$, we can extend the admissible range of speeds for which the bridge failure regime studied here is possible. Likewise, a similar effect can be achieved if $\varpi_{0}>1$. These values therefore define a rectangle (in general) in β–ϖ space within which a *semi-infinite bridge crack regime* can be found.

Outside this region, non-steady failure regimes can be encountered. Assuming $0<\varpi <\varpi_{0}$, i.e. horizontal links in the medium remain intact, for $\beta >\beta_{0}$, based on the analysis of figure 6 in §5d, it can be conjectured that the crack propagates with a more complex bridge fracture regime which we term the macro-cell bridge crack regime (figure 8). This fracture regime involves a bridge crack whose structure and periodicity change with increase of the initial stored potential energy in the system. This results from the fact that the increase in this energy allows more odd-numbered vertical nodes behind the crack to surpass the threshold for failure. The accumulation of this process, owing to the increase in stored initial energy, would allow transition to the regime where a *semi-infinite open crack* can propagate in the structure at a second critical value of β that we denote by $\beta_{1}$ (figure 8).

In a similar way, one can also consider regimes where $0<\beta <\beta_{0}$ and $\varpi >\varpi_{0}$. Analogously, we obtain descriptions of new fracture regimes. Possible candidates include

(i) a *finite bridge crack* regime for $\varpi_{0}<\varpi <\varpi_{1}$. Here, the bridge crack is accompanied by the appearance of failure sites along the horizontal chains. This causes the chain to partition at some point behind the crack front, leaving a semi-infinite chain in which there exists a finite bridge crack (figure 8). Note that within this regime, the macro-cell forming the finite bridge crack can vary with increasing γ. 
(ii) the complete failure of all horizontal links behind the fracture front when $\varpi >\varpi_{1}$, owing to the increase of initial stored energy in the system. Here, the mechanism for failure would be akin to that observed in the untangling of the so-called stick bomb [35], where the transition front in the medium creates a completely destroyed region behind it, with the fully intact region ahead of this point.

These regimes and potential β–ϖ subdomains are illustrated in figure 8, which, together with the blue curve presented there based on the analytical solution of [36], give us a way to predict possible steady and non-steady failure regimes. Finally, we note that for $\varpi >\varpi_{0}$ and $\beta >\beta_{0}$ (figure 8), one could encounter a range of complex failure regimes, possibly involving combinations of mechanisms already discussed above.

## 6. Conclusion

In this article we have studied the spontaneous failure of a discrete elastic chain [36] that occurs as a result of an initial stress imposed on the system. The failure mode considered involves a propagating bridge crack represented by the removal of every other vertical member of the system at a uniform speed. We have used the analytical solution from [36] characterizing this failure response to determine whether the considered steadily propagating regime can be realized. This solution, by construction, assumes that the medium will fail at the crack front. However, as shown in [36], this assumption is not valid for all crack speeds.

We have used the analytical solution to perform a study of the behaviour of horizontal and vertical links assumed to remain intact during the propagation of the bridge crack. As a consequence, we have shown that the solution serves to indicate regimes where links outside the location of the crack front can fail. This helps to determine limits on the speed for the bridge crack regime. Simultaneously, these limits represent the emergence of non-steady regimes, whose behaviour in time cannot be revealed by the solution that can accurately predict its onset.

Interestingly, these non-steady regimes appear as a result of critical elongations achieved by the lattice links behind the crack front. The physical reasoning behind this is that the bridge crack area is formed by periodically placed sites that are less constrained than the remaining regions of the medium. Nodes at these locations are therefore capable of larger displacements that are promoted by the release of waves into the structure as the bridge crack advances. In turn, these nodes promote greater displacements for their nearest neighbours, which are connected to intact vertical members of the structure. This allows such nodes to achieve the required critical displacement for failure.

In all regimes considered, the initiation of these non-steady regimes was never observed to occur as a result of unfeasible deformations ahead of the crack front. However, it is worth mentioning that waves propagating ahead of the crack may promote non-steady regimes. We note that this scenario typically occurs for low crack speeds that are seldom admissible but not impossible to realize [36]. In the analysis of non-steady failure processes, figure 8, which takes into account the structural integrity of the entire system, becomes important in identifying the potential structure and type of these regimes.

Our analysis also leads to new perspectives on possible novel non-steady regimes of failure within the chain. Simultaneously, it raises open questions about how newly identified regimes propagate. For instance, can one identify the strength ratio β for which a bridge crack can propagate that is formed from a sparse distribution of intact links in its wake? Additionally, what value of the strength ratio ϖ is required to promote the propagation of the failure phenomenon akin to the transition process observed in stick bombs [35]?

The tools developed here can be extended to study the influence of dynamic processes and loading on the failure of elastic structured systems through a range of complex mechanisms. Further, this work can be extended to the study of other multi-scale systems described by additional degrees of freedom, such as beam-made structures where rotations and moments play a role in their motion in addition to displacements and forces. We envision that the study presented here will have applications in the development of advanced materials and engineering assemblies and help to enhance their resilience to dynamic and static processes.

## Data Availability

This article has no additional data.

## References

[1] Slepyan LI. 1981 Dynamics of a crack in a lattice. Sov. Phys. Dokl. **26**, 538-540.

[2] Slepyan LI. 1981 Crack propagation in high-frequency lattice vibration. Sov. Phys. Dokl. **26**, 900-902.

[3] van Leewen JMJ. 2010 The domino effect. Am. J. Phys. **78**, 721-727. (10.1119/1.3406154)

[4] Noble B. 1958 Methods based on the Wiener-Hopf technique for the solution of partial differential equations. New York, NY: Pergamon Press.

[5] Slepyan LI. 2002 Models and phenomena in fracture mechanics. Berlin, Germany: Springer.

[6] Mishuris GS, Movchan AB, Slepyan LI. 2007 Waves and fracture in an inhomogeneous lattice structure. Waves Random Complex Media **17**, 409-428. (10.1080/17455030701459910)

[7] Nieves MJ, Movchan AB, Jones IS, Mishuris GS. 2013 Propagation of Slepyan’s crack in a non-uniform elastic lattice. J. Mech. Phys. Solids **61**, 1464-1488. (10.1016/j.jmps.2012.12.006)

[8] Berinskii IE, Slepyan LI. 2017 How a dissimilar-chain system is splitting: quasi-static, subsonic and supersonic regimes. J. Mech. Phys. Solids **107**, 509-524. (10.1016/j.jmps.2017.07.014)

[9] Gorbushin N, Mishuris G. 2019 Dynamic fracture of a dissimilar chain. Phil. Trans. R. Soc. A **377**, 20190103. (10.1098/rsta.2019.0103) 31474212 PMC6732372

[10] Piccolroaz A, Gorbushin N, Mishuris G, Nieves MJ. 2020 Dynamic phenomena and crack propagation in dissimilar elastic lattices. Int. J. Eng. Sci. **149**, 103208. (10.1016/j.ijengsci.2019.103208)

[11] Mishuris GS, Movchan AB, Slepyan LI. 2009 Localised knife waves in a structured interface. J. Mech. Phys. Solids **57**, 1958-1979. (10.1016/j.jmps.2009.08.004)

[12] Gorbushin N, Mishuris G. 2017 Analysis of dynamic failure of the discrete chain structure with non-local interactions. Math. Meth. Appl. Sci. **40**, 3355-3365. (10.1002/mma.4178)

[13] Mishuris GS, Movchan AB, Bigoni D. 2012 Dynamics of a fault steadily propagating within a structural interface. SIAM J. Multiscale Modell. Simul. **10**, 936-953. (10.1137/110845732)

[14] Gorbushin N, Mishuris G. 2018 Dynamic fracture of a discrete media under moving load. Int. J. Solids Struct. **130–131**, 280-295. (10.1016/j.ijsolstr.2017.09.026)

[15] Gorbushin N, Vitucci G, Mishuris G, Volkov G. 2017 Influence of fracture criteria on dynamic fracture in a discrete chain. Int. J. Fract. **209**, 131-142. (10.1007/s10704-017-0246-7)

[16] Mishuris GS, Movchan AB, Slepyan LI. 2008 Dynamics of a bridged crack in a discrete lattice. Quart. J. Mech. Appl. Math. **61**, 151-159. (10.1093/qjmam/hbm030)

[17] Slepyan LI, Ayzenberg-Stepanenko MV. 2002 Some surprising phenomena in weak-bond fracture of a triangular lattice. J. Mech. Phys. Solids **50**, 1591-1625. (10.1016/S0022-5096(01)00141-7)

[18] Brun M, Movchan AB, Slepyan LI. 2013 Transition wave in a supported heavy beam. J. Mech. Phys. Solids **61**, 2067-2085. (10.1016/j.jmps.2013.05.004)

[19] Brun M, Giaccu GF, Movchan AB, Slepyan LI. 2014 Transition wave in the collapse of the San Saba Bridge. Front Matter **1**, 12. (10.3389/fmats.2014.00012)

[20] Movchan AB, Brun M, Slepyan LI, Giaccu GF. 2015 Dynamic multi-structure in modelling a transition flexural wave. Mathematika **61**, 444-456. (10.1112/S0025579314000321)

[21] Nieves MJ, Mishuris GS, Slepyan LI. 2016 Analysis of dynamic damage propagation in discrete beam structures. Int. J. Solids Struct. **97–98**, 699-713. (10.1016/j.ijsolstr.2016.02.033)

[22] Garau M, Nieves MJ, Jones IS. 2019 Alternating strain regimes for failure propagation in flexural systems. Q. J. Mech. Appl. Math. **72**, 305-339. (10.1093/qjmam/hbz008)

[23] Ryvkin ML, Slepyan LI. 2010 Crack in a 2D beam lattice: analytical solutions for two bending modes. J. Mech. Phys. Solids **58**, 902-917. (10.1016/j.jmps.2010.03.006)

[24] Ryvkin M. 2012 Analytical solution for a Mode III crack in a 3D beam lattice. Int. J. Solid Struct. **49**, 2839-2847. (10.1016/j.ijsolstr.2012.04.003)

[25] Ryvkin M, Cherkaev A. 2021 Analysis of randomly damaged triangular beam lattice: elastic field and effective properties. Math. Mech. Solids **26**, 1219-1237. (10.1177/10812865211021637)

[26] Cherkaev A, Ryvkin M. 2019 Damage propagation in 2D beam lattices: 1. Uncertainty and assumptions. Arch. Appl. Mech. **89**, 485-501. (10.1007/s00419-018-1429-z)

[27] Cherkaev A, Ryvkin M. 2019 Damage propagation in 2D beam lattices: 2. Design of an isotropic fault-tolerant lattice. Arch. Appl. Mech. **89**, 503-519. (10.1007/s00419-018-1428-0)

[28] Livasov P, Mishuris G. 2019 Numerical factorization of a matrix-function with exponential factors in an anti-plane problem for a crack with process zone. Phil. Trans. R. Soc. A **377**, 20190109. (10.1098/rsta.2019.0109) 31474200 PMC6732375

[29] Marder M, Gross S. 1995 Origin of crack tip instabilities. J. Mech. Phys. Solids **43**, 1-48. (10.1016/0022-5096(94)00060-I)

[30] Slepyan LI, Movchan AB, Mishuris GS. 2010 Crack in a lattice waveguide. Int. J. Fract. **162**, 91-106. (10.1007/s10704-009-9389-5)

[31] Slepyan LI, Ayzenberg-Stepanenko MV, Mishuris GS. 2015 Forerunning mode transition in a continuous waveguide. J. Mech. Phys. Solids **78**, 32-45. (10.1016/j.jmps.2015.01.015)

[32] Nieves MJ, Mishuris GS, Slepyan LI. 2016 Transient wave in a transformable periodic flexural structure. Int. J. Solids Struct. **112**, 185-208. (10.1016/j.ijsolstr.2016.11.012)

[33] Gorbushin NA, Mishuris GS. 2017 Admissible steady-state regimes of crack propagation in a square-cell lattice. Mech. Solids **52**, 44-548. (10.3103/S0025654417050090)

[34] Mishuris GS, Slepyan LI. 2014 Brittle fracture in a periodic structure with internal potential energy. Proc. R. Soc. A **470**, 20130821. (10.1098/rspa.2013.0821) 24808756 PMC3973395

[35] Sautel J, Bourges A, Caussarieu A, Plihon N. 2017 The physics of a popsicle stick bomb. Am. J. Phys. **85**, 783-790. (10.1119/1.5000797)

[36] Ayzenburg-Stepanenko M, Mishuris G, Slepyan L. 2014 Brittle fracture in a periodic structure with internal potential energy. Spontaneous crack propagation. Proc. R. Soc. A **470**, 20140121. (10.1098/rspa.2014.0121) PMC3973395 24808756

